# Radiological Outcomes According to the Matta Score After the Surgical Fixation of Acetabular Fractures

**DOI:** 10.7759/cureus.74803

**Published:** 2024-11-30

**Authors:** Muhammad Younus Khan Durrani, Usman Ali, Zaigham Jamil, Masood Umer

**Affiliations:** 1 Orthopaedic Surgery, Aga Khan University Hospital, Karachi, PAK; 2 Surgery, Aga Khan University Hospital, Karachi, PAK; 3 Orthopaedic Surgery, The Aga Khan University, Karachi, PAK

**Keywords:** acetabular fractures, demographics, developing countries, fracture patterns, hospital stay, lmic-specific classification, matta criteria, orif, radiological outcomes

## Abstract

Background

Acetabular fractures, a rising concern in developing countries, pose a significant challenge due to their complexity and association with post-operative complications. Often caused by high-energy mechanisms like falls and motor vehicle accidents, these fractures require accurate reduction to prevent long-term issues and the potential need for hip replacement. This study investigates the radiological outcomes of acetabular fracture surgery at six months, focusing on the effectiveness of achieving anatomical reduction using the Matta criteria in a low-and middle-income country (LMIC) setting.

Methods and materials

This prospective study was conducted at a tertiary care center in Pakistan from May 2023 to December 2023, with ethical approval. Patients with isolated acetabular fractures were recruited. Preoperative X-rays and CT scans classified fractures using the Judet and Letournel Classification. Six-month postoperative X-rays were assessed using Matta radiographic criteria. Appropriate statistical analysis was deployed with a significance level at p < 0.05.

Results

A total of 33 cases met the study criteria, and the mean average age of patients was 44.18 ±17.2 years. Males constituted 87.9% of the cases. Longer hospital stays were associated with poorer outcomes (p < 0.001). Fracture patterns were significant predictors of outcomes (p < 0.001). Six months post-surgery, 45.5% of patients had excellent results, 24.2% had good results, and 15.2% each had fair and poor results according to the Matta radiographic criteria. Avascular necrosis (AVN) developed in 9.1% of patients. Of the 10 patients with femoral head dislocation, only one developed AVN

Conclusion

This LMIC-based study investigated factors affecting outcomes in patients with acetabular fractures treated using Open Reduction and Internal Fixation (ORIF). We found a relatively younger patient population, and injury patterns suggested a link to the local environment (e.g., traffic accidents). Optimizing hospital stay and timely surgery improved radiological outcomes as assessed by Matta criteria. While limitations exist, the study supports using Matta criteria in LMICs. Additionally, the use of plain radiographs, rather than CT scans, offers a cost-effective and radiation-reducing alternative for post-operative evaluation in resource-constrained settings.

## Introduction

Introduction

Acetabular fractures, primarily resulting from high-energy trauma are complex and challenging injuries. Treating these fractures has consistently posed significant challenges (complex anatomy, high vascularity, achieving and maintaining anatomical reduction) for orthopedic surgeons [1-3]. With an annual incidence of 8.1 per 100,000 persons these fractures are rare [4]. However, the incidence is increasing, particularly in developing countries [5]. The majority of acetabular fractures are caused by high-energy trauma, with 80.5% resulting from road accidents and 10.7% from falls from height [6]. Although less common, acetabular fractures can also result from low-energy falls, such as fragility fractures in osteoporotic patients [7-9].

These fractures are more often seen in young active patients and have been associated with a high rate of post-injury complications [10]. The displacement of the fracture fragments causes hip joint incongruency, leading to pain and an inability to bear weight, which results in functional disability. If left untreated, this can lead to necrosis of the femoral head and hip arthritis, necessitating second and third surgical interventions to prevent further complications [7]. It is a well-known fact that displaced fractures of the acetabulum are best treated with anatomical reduction and rigid internal fixation. [7] The main goals are joint surface congruency, stability, and early mobilization. However, the anatomical complexity of the hip joint makes it very difficult for reduction and internal fixation [7]. If accurate reduction (displacement of less than 3 mm) is achieved these complications can be prevented and the need for total hip arthroplasty can be delayed for approximately 25 years in 79% of the patients even in the elderly population [11-13]. On the contrary, poor reduction, fracture displacement of more than 2 mm, dislocation, and later surgery would likely have poor outcomes in terms of functionality and pain [14, 15]. 

Open Reduction and Internal Fixation (ORIF) of the acetabulum is a common procedure at our institute. The objective of this study is to determine the radiological outcomes of the acetabular fractures, six months post-procedure using the Matta radiographic criteria [7]. The Matta radiographic criteria provide a convenient, fast, and effective tool for assessing and grading the radiological outcomes of surgical repair of acetabular fractures.

Although numerous studies have evaluated outcomes following acetabular fracture repairs, our study addresses a critical gap by focusing on a low- and middle-income country (LMIC) setting. This is significant because the challenges and limitations in LMICs such as limited resources, varying levels of surgical expertise, and diverse patient demographics can profoundly influence treatment outcomes. [5] By providing data specific to an LMIC context, our study aims to offer insights that are currently underrepresented in literature.

This article was previously posted to the Research Square preprint server on August 27, 2024 (https://doi.org/10.21203/rs.3.rs-4979820/v1).

## Materials and methods

This prospective study was conducted at a tertiary care center hospital in one of the largest cities of Pakistan attracting a diverse population from all sorts of backgrounds. The study adhered to the principles of the Declaration of Helsinki. All the appropriate ethical approvals were obtained from Aga Khan University Hospital’s Institutional Review Board (IRB) committee (ERC reference number: 2022-0525-22307). Between May 2023 and December 2023, all patients presenting to the clinic or emergency department with acetabular fractures were recruited. Only patients with isolated acetabular fractures and those who gave consent for participation in the study were included, while those with concomitant femoral head injuries or other fractures, as well as those undergoing arthroplasty within six months of initial acetabular osteosynthesis, were excluded. Additionally, patients without a six-month follow-up and those initially operated outside Aga Khan University Hospital (AKUH) were excluded from recruitment.

Upon recruitment, preoperative X-rays and CT scans were meticulously reviewed to determine the type of fracture according to the Judet and Letournel Classification [16]. Subsequently, X-rays taken at the six-month postoperative follow-up were examined, and the Matta radiographic criteria were calculated based solely on radiological outcomes [14]. The collected data were tabulated and then thoroughly reviewed to generate the results presented in this paper.

During the review of X-rays, variables that could potentially influence the procedure's outcome and, consequently, the Matta radiographic criteria were also assessed. These variables included the types of fractures, the presence or absence of femoral head dislocation, and the visibility of intra-articular fragments in the preoperative radiographs.

The results were analyzed using descriptive statistics and comparisons among various groups. Categorical data were summarized as proportions and percentages, while discrete (quantitative) data were presented as means with standard deviations (SD).

## Results

A total of 33 cases met the criteria for this study. The mean average age of patients was 44.18 ±17.2 years. years. Age distribution did not show a statistically significant relationship with injury severity (p = 0.509), with individuals aged 25-30 and those over 50 years old comprising the majority of cases. Gender also exhibited a notable correlation (p < 0.001), with males (87.9%) constituting a significantly higher proportion of injuries compared to females (12.1%). Length of hospital stay (LOS) demonstrated a strong association with outcomes, with a longer LOS favoring poorer outcomes. Detailed descriptive and analytical analyses of the variables are given in Table 1 and Table 2, respectively.

**Table 1:** Radiological outcomes using Matta score Chi-square test were used to determine associations between the above categorical variables and outcomes. ASA: American Society of Anesthesiologists; LOS: Length of Stay; RTA: Road Traffic Accident

Variable	Excellent	Good	Fair	Poor	Total	P value	Chi-square value
Age						0.509	5.27
25-30	11	4	3	2	20		
<25	0	1	0	0	1		
>50	5	2	2	3	12		
Gender							
						<0.001	12.93
Male	15	7	5	2	29		
Female	1	0	0	3	4		
LOS						0.256	4.05
<7	10	2	2	1	15		
>7	6	5	3	4	18		
ASA Grade						0.766	5.74
1	5	2	2	1	10		
2	5	3	1	2	11		
3	6	1	2	1	10		
4	0	1	0	1	2		
Days between injury						0.048	7.90
<7	15	6	3	2	26		
>7	1	1	2	3	7		
Fracture Pattern						<0.001	6.92
Simple	11	2	4	1	18		
Associated	5	5	1	4	15		
Source of injury						0.428	5.96
Fall	3	1	1	1	6		
RTA	13	6	4	3	26		
Twisting of leg	0	0	0	1	1		
Dislocation						0.133	5.60
Yes	3	1	3	3	10		
No	13	5	2	2	23		
Avascular Necrosis						<0.001	18.48
Yes	0	0	0	3	3		
No	16	7	5	2	30		

**Table 2:** Descriptive statistics for qualitative variables ASA: American Society of Anesthesiologists; LOS: Length of Stay; RTA: Road Traffic Accident

Variable	%/ mean ± SD
Age	44.18 ±17.2
25-30	20 (60.61)
<25	1 (3.03)
>50	12 (36.36)
Gender	
Male	29 (87.88)
Female	4 (12.12)
LOS	9.45±6.58
<7	12 (36.36)
>7	21 (63.34)
ASA Grade	
1	10 (30.30)
2	11 (33.33)
3	10 (30.30)
4	2 (6.06)
Days between injury	Average 4.6
<7	26 (78.79)
>7	7 (21.21)
Source of injury	
Fall	6 (18.18)
RTA	26 (78.79)
Twisting of leg	1 (3.03)
Dislocation	
Yes	10 (31.25)
No	22 (68.75)

The frequencies of fracture patterns, identified according to the Judet and Letournel classification for acetabular fractures are summarized in Table 3 and Table 4. Fracture pattern emerged as a significant predictor of outcome (p < 0.001), with associated fractures demonstrating a higher likelihood of poor outcomes compared to simple fractures (Table 1).

**Table 3:** Frequencies of fracture patterns

Fracture Pattern	
Simple	15 (45.45%)
Only Posterior wall	8 (24.24%)
Anterior column	3 (9.09%)
Transverse	2 (6.06%
Only Posterior column	1 (3.03%)
Only Anterior wall	1 (3.03%)
Associated	18 (54.55%)
Posterior Wall + Posterior column	5 (15.15%)
Both column	5 (15.15%)
Transverse + Posterior Wall	4 (12.12%)
Anterior wall + Posterior wall + Hemi transverse	3 (9.09%)
Transverse and vertical	1 (3.03%)

**Table 4:** Matta Score according to type of fracture

Type of Fracture	Excellent	Good	Poor	Fair	Grand Total
Posterior Wall	3	2	2	1	8
Posterior Wall + Posterior Column	5	-	-	-	5
Both Column Fracture	2	1	1	1	5
Transverse + Posterior Wall	2	-	-	2	4
anterior column fracture	1	1	1	-	3
Anterior column + posterior hemi transverse	1	1	-	1	3
Transverse Fracture	1	-	1	-	2
Anterior Wall Fracture	-	1	-	-	1
T shaped	1	-	-	-	1
Posterior Column Fracture	-	1	-	-	1
Grand Total	16	7	5	5	33

**Figure 1:**
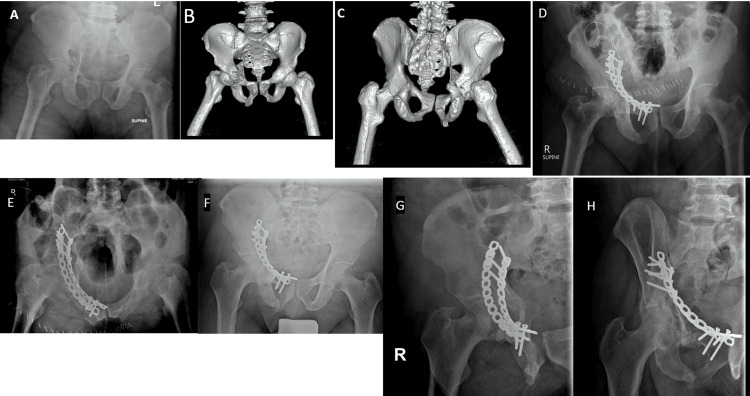
Radiological progression of a case with excellent outcome: initial presentation, post-operative (6 weeks), and 6-month follow-up images The figure shows a sequence of radiological images of a 58-year-old male patient who presented following a motor vehicle accident. Fig. A: X-rays taken on presentation, revealing an anterior with posterior hemi-transverse fracture. Fig. B and C: CT scan 3D reconstruction images providing detailed visualization of the fracture. ORIF (Open Reduction and Internal Fixation) was performed. Fig. D and E: X-rays taken post-operatively, showing the initial results of the surgical intervention. Fig. F, G, and H: X-rays taken 6 months post-operatively, illustrating the progression of bone healing and the patient's condition over time.

**Figure 2:**
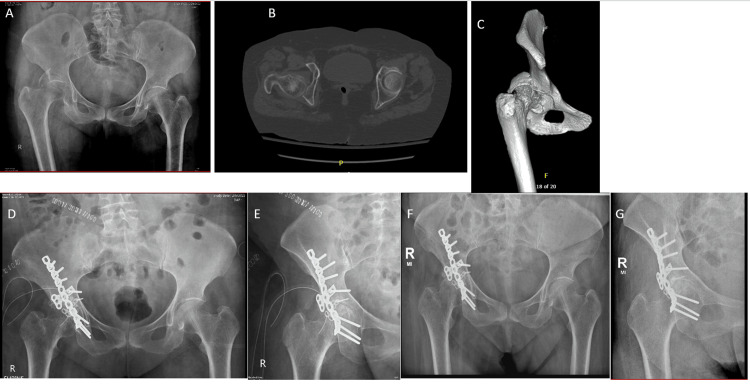
Radiological progression of a 60-year-old male with good outcome: initial presentation, post-operative (6 weeks), and 6-month follow-up images A 60-year-old male presented following a car-on-car road traffic accident, resulting in a right acetabulum fracture with involvement of the anterior column. Fig. A: Initial X-ray revealing a right acetabulum fracture involving the anterior column. Fig. B: Obturator view X-ray of the right acetabulum confirming the anterior column fracture. Fig. C: Axial CT scan of the hip showing a clear anterior column fracture. Fig. D: Sagittal CT scan demonstrating the same anterior column fracture. Fig. E: 3D reconstruction from a CT scan providing detailed visualization of the anterior column fracture. Fig. F: Postoperative X-ray showing the fixation of the fracture. Fig. G: X-ray taken 6 months post-fixation, assessing the healing and alignment of the fracture

**Figure 3:**
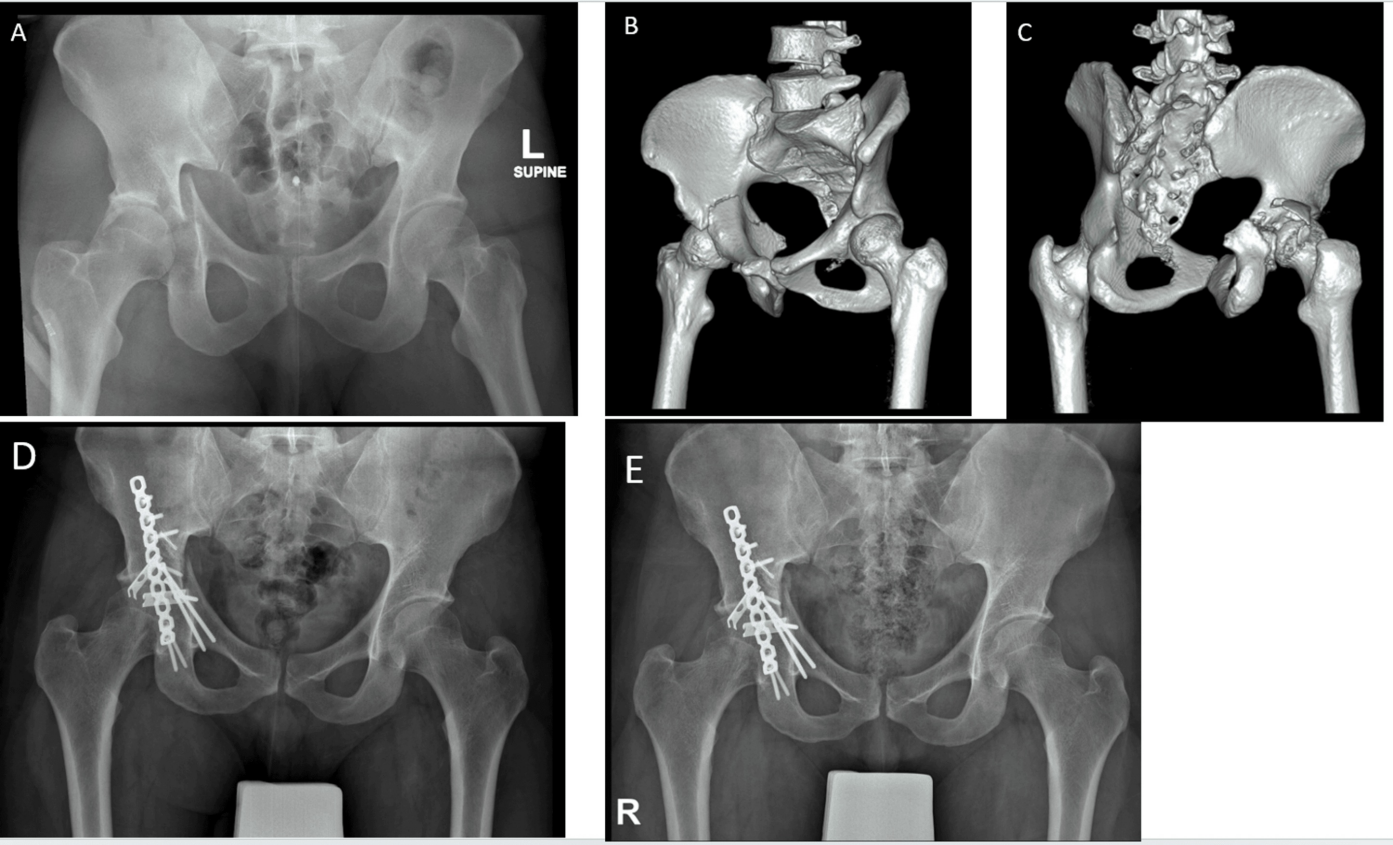
Radiological progression of a case with fair outcome: initial presentation, post-operative (6 weeks), and 6-month follow-up image The figure shows a sequence of radiological images of a 35-year-old patient who presented following a motor vehicle accident. Fig. A: X-rays taken on presentation, revealing a transverse + posterior wall fracture. Fig. B and C: CT scan 3D reconstruction images showing the fracture in greater detail. ORIF (Open Reduction and Internal Fixation) was performed. Fig. D: X-rays taken 6 weeks post-operatively, demonstrating initial healing and hardware placement. Fig. E: X-rays taken 6 months post-operatively, illustrating the patient’s condition and the progression of bone healing.

**Figure 4:**
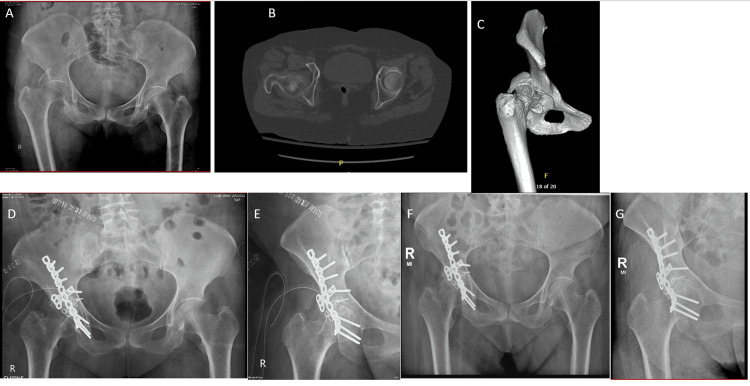
Radiological progression of a 68-year-old female with poor outcome: initial presentation, post-operative (6 weeks), and 6-month follow-up images A 68-year-old female presented following a car accident with a poor outcome. Initial imaging confirmed a right acetabulum fracture with a posterior wall fracture. Fig. A: Initial X-ray showing a right acetabulum fracture with a posterior wall fracture. Fig. B: CT scan axial view of the hip, indicating a posterior wall fracture with posterior subluxation of the femoral head. Fig. C: 3D reconstruction CT scan, providing a detailed view of the fracture and associated dislocation. Fig. D: AP view X-ray of the pelvis on postoperative day 1, demonstrating successful reduction and fixation. Fig. E: Obturator view of the right hip postoperatively, showing restoration of the articular surface and reduced femoral head. Fig. F: AP view of the pelvis 5 months postoperatively, depicting reduced joint space. Fig. G: Obturator view of the pelvis 5 months postoperatively, showing maintained reduction but reduced joint space.

**Figure 5:**
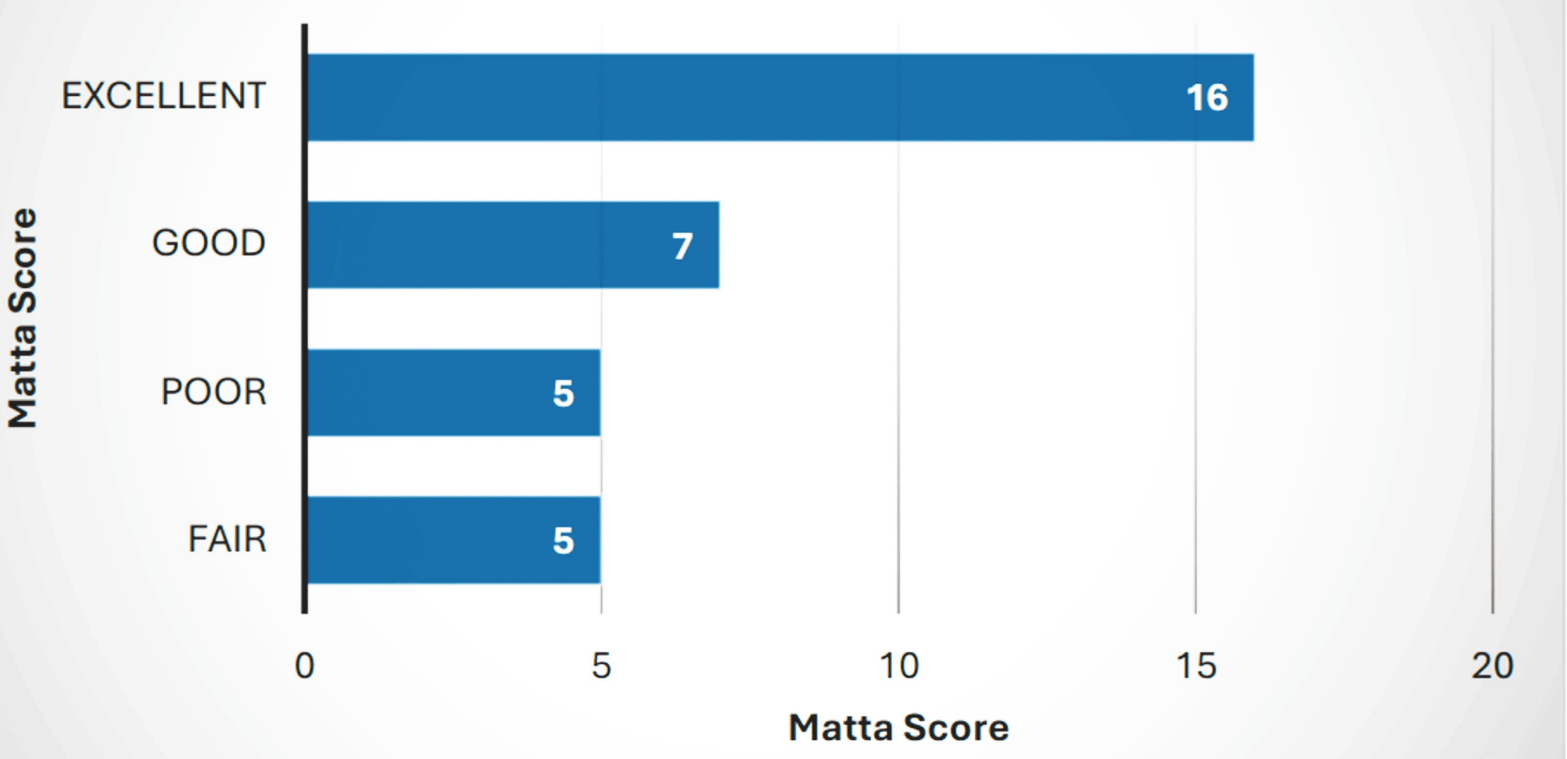
Radiological Matta Scores A horizontal bar graph displaying the distribution of Matta scores, ranging from excellent to fair, among the study cohort. Each bar represents the frequency of patients falling within different score categories, aiding in the visualization of Matta radiographic criteria after surgical fixation of acetabular fracture.

Six months post-surgery, follow-up radiological images were reviewed. Figures 1-4 present detailed case examples. According to the Matta radiographic criteria, 15 (45.5%) of the operated cases had excellent outcomes, eight (24.2%) had good results, and five (15.2%) each had fair and poor results (Figure 5). Additionally, three patients (9.1%) developed avascular necrosis (AVN) at the six-month post-surgery follow-up.

Ten patients presented with femoral head dislocation. Within this cohort, only one patient developed avascular necrosis (AVN) within six months post-fixation. Among the subset of patients who developed AVN, one exhibited anterior column fracture, another manifested a posterior wall fracture, and the third exhibited a transverse fracture.

## Discussion

Acetabular fractures pose a significant challenge, influenced by patient factors (comorbidities and injury mechanisms) and surgeon-related considerations (including patient selection and surgical timing). Our meticulous study analyzed 33 patients treated for acetabular fractures using open reduction and internal fixation (ORIF). Postoperative radiological assessments consistently demonstrated significantly improved outcomes, aligning with prior research. Notably, gender played a pivotal role, with males experiencing a higher proportion of injuries. Additionally, a longer hospital stay favored poorer results. Furthermore, fracture patterns significantly impacted outcomes, with associated fractures showing a higher likelihood of unfavorable results compared to simple fractures.

Our findings demonstrate a younger patient population for acetabular fractures, with a mean age of 43.2 years. While no significant association was established with age, the data highlights a predominance of younger individuals, particularly those aged 25-30. This aligns with previous research suggesting acetabular fractures predominantly impact younger demographics [17-18]. The relatively lower mean age in our study compared to other populations might be attributed to the higher proportion of young individuals residing in this specific region compared to Western countries.

In line with other regional studies, our investigation primarily involved a male population, with 87.9% of patients being male [19] This gender predominance can be attributed to the high incidence of acetabular injuries resulting from road traffic accidents (RTAs). Our analysis revealed that over 75% of these injuries occurred due to RTAs. The local socio-environmental context likely contributes to this disparity, as males are more frequently exposed to street-related risks. In contrast, females tend to experience low-energy trauma associated with osteoporosis.

Optimizing the length of hospital stay (LOS) is essential for enhancing patient outcomes, as previous studies suggest [20]. While our study did not reach statistical significance, it did reveal a notable trend indicating that longer LOS is associated with poorer radiological outcomes. This pattern may reflect underlying risks linked to extended hospital stays, such as a heightened likelihood of hospital-acquired infections, complications from prolonged immobilization, and other adverse factors. These findings underscore the importance of monitoring LOS to potentially mitigate these risks and improve recovery trajectories.

Our study employed the Matta radiographic criteria to evaluate post-operative outcomes, aligning with previous research demonstrating its effectiveness [21] Achieving anatomic reduction in acetabular fractures is crucial to minimize complications like avascular necrosis and arthritis, as evidenced by the decrease in osteoarthritis incidence from 31% to 14% due to improved surgical techniques and reduction quality [8]. Even if future arthroplasty is planned, a suboptimal initial reduction can complicate the procedure [22]. Conversely, achieving anatomic reduction can potentially eliminate the need for secondary hip procedures, with studies reporting a 70% success rate [13, 23]. Our results mirrored these findings, with 45.5% of patients experiencing excellent outcomes and 24.2% experiencing good outcomes at six months post-surgery. The avascular necrosis rate (9.09%) was comparable to Islam et al.'s study (8.0%), further emphasizing the importance of reduction for optimal patient outcomes [24] Similar studies by Charan et al. and Kizkapan et al. also utilized the Matta criteria and reported favorable outcomes with proper reduction [9, 25]. These findings collectively support the effectiveness of the Matta criteria in assessing post-operative acetabular fracture reduction and its impact on long-term patient outcomes.

In the context of LMICs, the use of plain radiographs over Computed Tomography (CT) scans offers several key advantages. X-rays are significantly more cost-effective and also reduce radiation exposure compared to CT, making them accessible to a broader patient population, an important consideration in resource-limited settings. By utilizing this approach, our study not only highlights the feasibility of achieving reliable assessments with available resources but also underscores its practical value in LMICs, where access to advanced imaging may be limited

Limitations of the study

While our study provides valuable insights into the factors influencing outcomes of acetabular fracture treatments, it is not without limitations. The relatively small sample size of 33 patients may limit the generalizability of our findings. A larger study would yield more robust results and strengthen the observed associations. Conducted in a single center, our study offers an in-depth understanding of the local demographic and socio-environmental factors, though it may not reflect variations in other areas. Including data from multiple centers would provide a broader perspective on patient characteristics and outcomes. Finally, we primarily relied on radiological outcomes using the Matta criteria. While valuable, incorporating functional outcome measures would provide a more comprehensive picture of patient recovery. Moreover, while the Letournel and Judet classification is popular and valuable, it overlooks key factors such as comminution and dislocation, which can significantly impact surgical planning and patient outcomes, often necessitating the use of preoperative CT scans for a more comprehensive assessment.

## Conclusions

In conclusion, our study investigated factors influencing outcomes in patients with acetabular fractures treated using open reduction and internal fixation (ORIF). The findings highlight the importance of patient demographics, particularly a younger patient population in our region compared to others. Additionally, gender and injury mechanisms (predominantly road traffic accidents) appear to play a role, potentially reflecting the local socio-environmental context. Furthermore, our study underscores the critical role of optimizing the length of hospital stay and timely surgical intervention for achieving better radiological outcomes.

The study supports the effectiveness of the Matta criteria in evaluating post-operative reduction and its influence on patient outcomes. However, limitations exist, including sample size, single-center nature, and reliance on radiological outcomes. Future research with larger, prospective, multicenter designs incorporating functional outcomes can further strengthen these findings.
